# Comparison of Weekly and Triweekly Cisplatin Regimens in the Treatment of Head and Neck Cancer: A Systematic Review and Meta-Analysis

**DOI:** 10.3390/cancers17091444

**Published:** 2025-04-25

**Authors:** Sylwester M. Kloska, Anna Kloska

**Affiliations:** Faculty of Medicine, Bydgoszcz University of Science and Technology, 85-796 Bydgoszcz, Poland; anna.kloska@pbs.edu.pl

**Keywords:** chemoradiotherapy, cisplatin, head and neck cancer, high dose, HNSCC, low dose

## Abstract

Background: Cisplatin-based chemoradiotherapy is the standard treatment for locally advanced head and neck squamous-cell carcinoma (LA-HNSCC); however, the optimal dosing regimen remains debated. This systematic review and meta-analysis aimed to compare treatment compliance, therapeutic efficacy, and toxicity profiles between weekly (30–50 mg/m^2^) and triweekly (100 mg/m^2^ every three weeks) cisplatin regimens in patients receiving concurrent radiotherapy for LA-HNSCC. Methods: A systematic literature search was conducted in PubMed, Google Scholar, and ClinicalTrials.gov to identify prospective clinical trials published before 16 January 2025, comparing weekly and triweekly cisplatin regimens. Studies were included if they reported treatment compliance, efficacy, and chemotherapy-related toxicities. Single-arm studies were excluded. Data extraction was performed independently by two reviewers, and the risk of bias was assessed using the Cochrane Risk of Bias (RoB) tool. Odds ratios (ORs) and 95% confidence intervals (CIs) were calculated for the primary endpoints: overall survival (OS) and chemotherapy completion rates. Heterogeneity was assessed using the I^2^ statistic. Results: Fifteen prospective clinical trials with 1572 patients (775 weekly and 797 triweekly) were included. Treatment compliance was similar between the regimens, with 74.76% (weekly) vs. 72.29% (triweekly) completing chemotherapy (*p* = 0.38). The mean cumulative cisplatin dose was significantly higher in the triweekly regimen (287.52 mg/m^2^ vs. 241.74 mg/m^2^, *p* = 0.04); however, the proportion of patients receiving a cumulative dose ≥200 mg/m^2^ did not differ significantly (*p* = 0.23). The therapeutic efficacy was comparable, with complete response rates of 63.18% (weekly) and 67.13% (triweekly) (*p* = 0.32) and OS rates at 2 years of 51.24% and 49.47% (*p* = 0.45). No significant differences were observed in the toxicity rates (any grade or grade ≥ 3) or mortality. The I^2^ statistic indicated insignificant heterogeneity across the studies. Interpretation: The results do not provide definitive evidence favoring one regimen over the other. Both regimens remain viable treatment options with comparable efficacy and adherence. Treatment selection should be individualized, considering toxicity risk, patient tolerability, and clinical factors.

## Simple Summary

Locally advanced head and neck squamous-cell carcinoma (LA-HNSCC) is often treated with concurrent chemoradiotherapy. A commonly used chemotherapy drug, cisplatin, can be given either once a week in smaller doses (30–50 mg/m^2^) or once every three weeks in a higher dose (100 mg/m^2^), but it is still unclear which schedule works better and causes fewer side effects. In this study, we carefully analyzed data from 15 clinical trials that included over 1500 patients to compare the weekly and three-weekly treatment schedules. We investigated and compared treatment compliance, treatment response, survival rates, and the occurrence of side effects. We found that both schedules were similar in terms of treatment success and side effects. Although patients receiving the three-weekly treatment received a higher total dose of cisplatin, this did not influence efficacy or safety outcomes. Overall, both schedules appear to be equally effective and safe. Our findings indicate that the treatment plan should be based on the individual patient’s needs, health condition, convenience, and ability to tolerate the treatment. These findings can help guide more personalized care for patients with advanced head and neck cancers.

## 1. Introduction

Cisplatin is one of the key chemotherapeutic agents used to enhance the effectiveness of radiotherapy in the treatment of locally advanced head and neck squamous-cell carcinoma (LA-HNSCC). As a platinum-based DNA-damaging agent, cisplatin exerts its cytotoxic effects by inducing DNA crosslinking, thereby inhibiting replication and promoting apoptosis in rapidly proliferating tumor cells [1]. When administered concurrently with radiotherapy, cisplatin acts as a radiosensitizer, enhancing the tumoricidal effects of ionizing radiation. This combined approach has been shown to improve locoregional control and overall survival in patients with HNSCC [2,3]. However, the optimal dosing schedule remains a subject of ongoing debate, with weekly low-dose and triweekly high-dose regimens being the most commonly utilized strategies [4,5,6,7,8,9].

Previous studies indicate that a cumulative cisplatin dose exceeding 200 mg/m^2^ is desirable and associated with improved therapeutic outcomes in patients with LA-HNSCC [10,11]. However, treatment-related toxicity and its severity remain the primary reasons for discontinuation of therapy. Evidence suggests that a significant proportion of patients receiving the standard high-dose cisplatin regimen of 100 mg/m^2^ every three weeks struggle with treatment-related adverse effects that prevent them from completing the three planned cycles of chemotherapy [12,13]. The most frequently reported and well-documented toxicities include mucositis and hematologic complications [14,15,16].

In response to these challenges, efforts have been made to develop alternative treatment regimens that reduce toxicity while maintaining therapeutic efficacy. One clinically accepted approach is the administration of weekly cisplatin at a dose of 40 mg/m^2^ over 6–7 weeks. The rationale behind this regimen is that it enhances treatment adherence by minimizing delays due to toxicity-related interruptions while also reducing both acute and long-term side effects [17,18]. Additionally, weekly cisplatin administration offers greater flexibility, allowing for treatment adjustments based on individual patient tolerability and the development of adverse effects.

Previous meta-analyses, including the comprehensive work by Szturz et al. [19], have compared weekly and triweekly cisplatin regimens in patients with HNSCC across a broad range of study types, including both prospective and retrospective designs as well as single-arm trials. While their findings have provided important insights into the comparative efficacy and toxicity profiles of low-dose and high-dose cisplatin, the inclusion of heterogeneous study designs may have introduced potential biases and limited the strength of direct comparisons between regimens. In particular, their analyses reported that weekly cisplatin was associated with lower rates of certain toxicities in definitive treatment settings but not in the postoperative context.

In contrast, the present systematic review and meta-analysis is, to our knowledge, the first to include only prospective two-arm clinical trials directly comparing weekly (30–50 mg/m^2^) and triweekly (100 mg/m^2^) cisplatin regimens administered concurrently with radiotherapy in LA-HNSCC. By focusing exclusively on head-to-head comparisons within controlled settings, this study aimed to reduce methodological bias and enhance the reliability of pooled estimates of efficacy, treatment compliance, and toxicity.

The objective of this systematic review and meta-analysis was to consolidate existing knowledge on the use of cisplatin in the treatment of LA-HNSCC when administered in either a weekly or triweekly regimen in combination with concurrent radiotherapy. This analysis focused on three key aspects: (a) patient adherence to the planned treatment cycle, (b) therapeutic efficacy, and (c) treatment-related toxicity. The hypothesis was that weekly cisplatin administration would be associated with a significantly lower incidence of high-grade toxicities while maintaining a therapeutic efficacy comparable to the standard triweekly regimen in patients with LA-HNSCC. By systematically evaluating and comparing these factors across the available studies, this review provides evidence-based insights that can support oncologists in making informed treatment decisions for patients with LA-HNSCC.

## 2. Materials and Methods

### 2.1. Search Strategy

This review was performed in accordance with the PRISMA (Preferred Reporting Items for Systematic Reviews and Meta-Analyses) guidelines [20] and was retrospectively registered in the PROSPERO international prospective register of systematic reviews under the registration number CRD420251032694. Registration was completed prior to the submission of the revised manuscript, in accordance with a recommendation provided by peer reviewers.

A systematic literature search was conducted to identify prospective clinical studies published before 16 January 2025 evaluating the efficacy and safety of chemotherapy regimens in patients with LA-HNSCC. The analysis focused on studies comparing weekly cisplatin administration at doses that did not exceed 50 mg/m^2^ with high-dose cisplatin administered every three weeks at 100 mg/m^2^, both in combination with concurrent radiotherapy. Studies were eligible for inclusion regardless of whether patients were treated in a definitive setting or had undergone surgical resection of a treatment-naïve tumor followed by postoperative treatment.

The systematic literature search was conducted using a predefined set of keywords to ensure comprehensive identification of relevant studies in the following databases and websites: PubMed, Google Scholar, and ClinicalTrials.gov. The search strategy included the following keyword combinations: (cisplatin OR cisplatinum) AND (head and neck cancer OR HNSCC OR Head and Neck Squamous Cell Carcinoma) AND (weekly OR triweekly OR 3-weekly OR 3 weekly OR every 3 weeks OR every three weeks). The search on Google Scholar was limited to the first 300 results ranked by relevance, as the search engine’s algorithm tends to generate a large number of results, including less relevant publications. In addition to the systematic search, we also performed citation tracking of relevant articles to identify additional studies that met the inclusion criteria.

Studies were excluded if they met any of the following criteria: (a) studies published before 1 January 2000; (b) studies not available in English; (c) studies including drugs or compounds other than cisplatin in the treatment regimen; (d) studies involving induction chemotherapy; (e) single-arm studies (only a weekly or triweekly arm); (f) preclinical studies, including in vitro experiments, animal models, and mathematical modeling studies; (g) non-primary research articles, such as reviews, secondary analyses, post hoc analyses, editorials, comments, letters, corrections, and surveys; (h) retrospective studies, case studies and reports, study protocols, meta-analyses, and registry-based studies; (i) studies investigating diseases or conditions other than LA-HNSCC; and (j) studies that did not report safety and/or efficacy outcomes. An automated screening step based on conditional formatting in Microsoft Excel version 2412 (Microsoft Corporation, Microsoft 365) was utilized to assist in the preliminary exclusion of ineligible records. This approach allowed the authors to flag records that met predefined exclusion criteria using rule-based filters applied to metadata fields such as the publication year, title keywords, and language. Specifically, formatting rules were designed to identify and highlight studies that met exclusion criteria (a)–(i). This automated filtering step supported more efficient screening but did not replace manual review.

Two authors independently assessed each publication for its eligibility for inclusion in this meta-analysis. In cases of discrepancies between their assessments, a thorough discussion was held to reach a final decision. This discussion included, among other aspects, determining whether the publication failed to meet any of the exclusion criteria.

### 2.2. Data Extraction

Data extraction was performed by one of the authors and subsequently reviewed by a second author to minimize the risk of errors. From the eligible publications, the following information was extracted: study characteristics (author, year, country, and study design); patient demographics (number of patients, age, sex, smoking history, and disease-related characteristics); treatment regimens (chemotherapy and radiotherapy protocols, including drug regimens, dosages, schedules, and any modifications); and treatment outcomes, encompassing both efficacy and adverse events. Efficacy measures such as locoregional control, overall survival, and progression-free survival were recorded. Adverse event data were collected and grouped into hematological and non-hematological complications. Each toxicity category was evaluated in terms of frequency and severity, with stratification into any grade and grade ≥ 3. In cases where a publication did not report a cumulative value for grade ≥ 3 toxicity but instead provided separate data for each toxicity grade, the frequencies of grades 3 and higher (grades 3; 4; and, if applicable, 5) were summed to obtain a total value for grade ≥ 3 toxicity. In cases where nausea and vomiting were reported separately, the higher values were selected for analysis. This approach allowed for a standardized comparison across studies. Summary data were extracted from published reports, and individual patient-level data were not requested from study authors. If a specific feature was not reported in a publication, its result was not recorded, and no extrapolation was performed.

The risk of bias in the included studies was assessed using the Cochrane Risk of Bias (RoB) tool for randomized trials. Two independent reviewers (S.M.K. and A.K.) evaluated each study. The RoB assessment results are available in the Supplementary Materials. Any disagreements were resolved by consensus. Automation tools were not used in the risk-of-bias assessment.

### 2.3. Outcomes

The primary endpoints of the analysis were overall survival at 2 years and chemotherapy completion rates. Secondary endpoints included other metrics related to the radiotherapy and chemotherapy protocols, treatment response, and treatment-related toxicities, including acute adverse events of any grade and grade ≥ 3.

### 2.4. Statistical Analysis

Two complementary statistical approaches were employed in this study: descriptive group-level comparisons and formal meta-analytic pooling for primary outcomes.

First, to compare continuous variables (e.g., the cumulative cisplatin dose and the number of chemotherapy cycles completed) between the weekly and triweekly regimens, the Shapiro–Wilk test was applied to assess normality. Depending on the distribution, group comparisons were performed using the two-sample Student’s *t*-test, with Welch’s correction applied when the assumption of equal variances was not met. A *p*-value < 0.05 was considered statistically significant for all tests.

For the two primary endpoints of the meta-analysis, 2-year overall survival (OS) and completion of planned chemotherapy, odds ratios (ORs) with corresponding 95% confidence intervals (CIs) were calculated for each study. Pooled estimates were computed using a random-effects model, applying inverse-variance weighting. In cases where an OR calculation would involve division by zero, the Haldane–Anscombe correction was applied, adding 0.5 to each cell, in accordance with Weber F. et al. [21].

The heterogeneity between the studies was assessed using the I^2^ statistic, which quantifies the proportion of total variability due to heterogeneity rather than chance. The pooled results are presented using forest plots, which include study-specific weights, confidence intervals, and summary estimates.

All analyses were performed using Microsoft Excel version 2412 (Microsoft Corporation, Microsoft 365), MedCalc Statistical Software version 23.1.6 (MedCalc Software bv, Ostend, Belgium; https://www.medcalc.org; 2025), and Google Colaboratory (2025, Google, available at https://colab.research.google.com/).

## 3. Results

**Figure 1:**
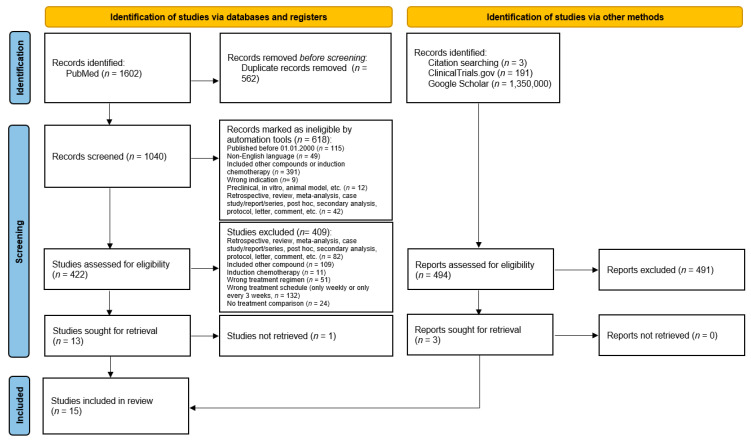
A PRISMA 2020 flow diagram. Conditional formatting in Microsoft Excel version 2412 (Microsoft Corporation, Microsoft 365) was used as an automation tool to identify ineligible publications based on their titles. Source: [20].

A study retrieval flow diagram is presented in Figure 1. Fifteen prospective clinical trials where patients were treated with single-agent cisplatin and concurrent radiotherapy were eligible for a meta-analysis comparing weekly and triweekly cisplatin treatment schedules. A summary of the included studies is presented in Table 1. Thirteen of these studies were randomized. The other two did not specify whether they were randomized.

### 3.1. Patient Characteristics

This meta-analysis included a total of 1572 patients, with 775 receiving weekly cisplatin and 797 receiving triweekly cisplatin. The majority of the patients in both groups were male. The mean ages of the patients were 54.8 and 53.9 years in the weekly and triweekly arms, respectively. The patient populations were generally comparable in terms of their age distributions, suggesting that differences in treatment outcomes were unlikely to be attributed to significant age-related variations. Disease staging was available for most studies, with the patients predominantly presenting with stage III or IV disease. The distributions of the primary tumor sites were similar in both groups. The most frequently affected regions were the oropharynx, oral cavity, and salivary glands, followed by the larynx and hypopharynx. Nasopharyngeal, nasal cavity, and paranasal sinus tumors were less commonly represented. Similarities between the patients receiving the weekly and triweekly cisplatin regimens minimized potential confounding effects and allowed for a more reliable comparison of treatment-related toxicities and efficacy. Detailed patient data are presented in the Supplementary Materials (Table S1).

### 3.2. Compliance with Chemotherapy Protocol

**Figure 2:**
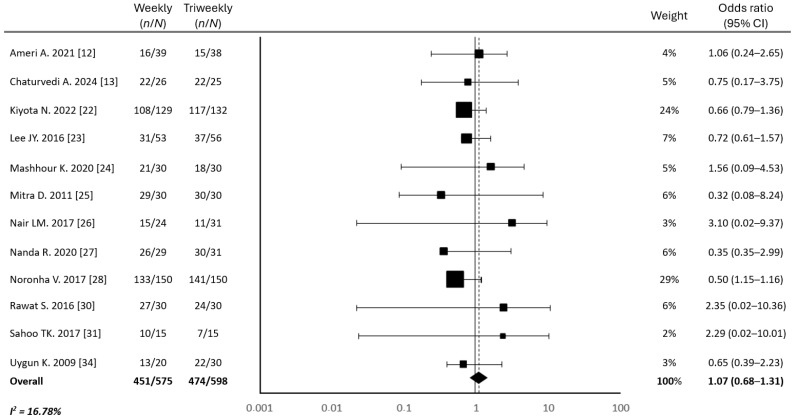
Forest plot of odds ratios for chemotherapy completion in weekly and triweekly cisplatin regimens. Abbreviations: CI, confidence interval; *n*, number of patients who completed chemotherapy; *N*, number of patients ([12,13,22,23,24,25,26,27,28,30,31,34]).

**Figure 3:**
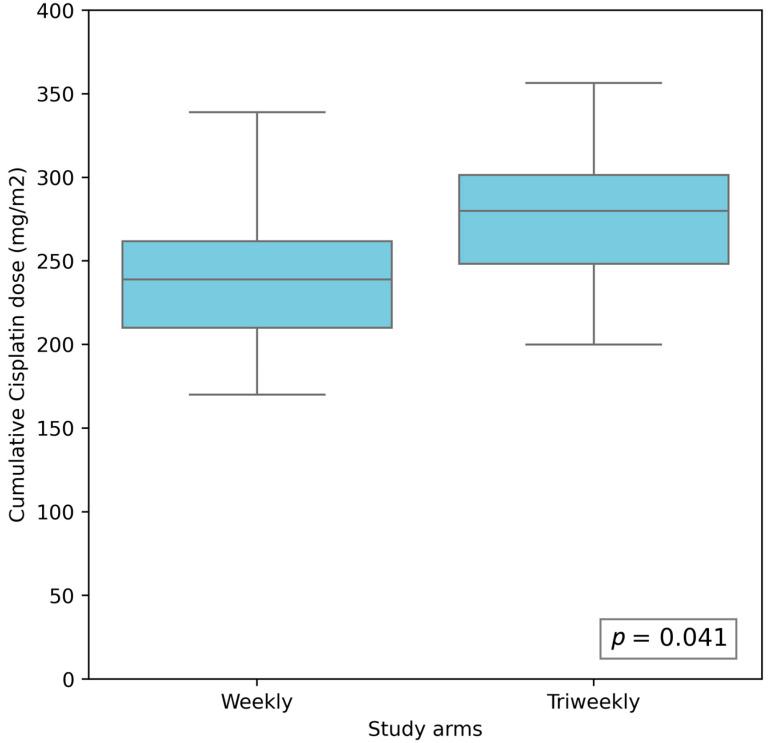
The cumulative cisplatin doses in the weekly and triweekly cisplatin regimens. The mean cumulative doses were 241.74 mg/m^2^ for the weekly regimen and 287.52 mg/m^2^ for the triweekly regimen.

There was no significant difference in treatment adherence between the two groups (Table 2). The median numbers of completed cycles were six and three for the weekly and triweekly cisplatin regimens, respectively. On average, 74.76% of the patients in the weekly cisplatin group and 72.29% of the patients in the triweekly cisplatin group completed the planned chemotherapy regimens (*p* = 0.38) (Figure 2). The mean cumulative cisplatin doses were 241.74 mg/m^2^ for the weekly regimen and 287.52 mg/m^2^ for the triweekly regimen, with a significantly higher cumulative dose observed in the triweekly group (*p* = 0.04) (Figure 3). Additionally, the proportions of patients receiving a cumulative cisplatin dose ≥200 mg/m^2^ were 70.64% and 78.75% in the weekly and triweekly groups, respectively (*p* = 0.23). These results suggest there was no significant difference between the two regimens in terms of reaching the desired cumulative dose threshold.

Overall, the findings indicate that both cisplatin regimens demonstrated comparable rates of treatment completion and comparable proportions of patients receiving an adequate cumulative dose of ≥200 mg/m^2^. However, the patients receiving the triweekly regimen received a significantly higher total cumulative cisplatin dose, which may have implications for treatment efficacy and toxicity.

### 3.3. Compliance with Radiotherapy Protocol

The total planned radiation doses across the analyzed studies ranged from 66 Gy to 70 Gy, consistent with standard therapeutic protocols. Ten studies reported which radiotherapy techniques were included in the treatments. They were Intensity-Modulated Radiation Therapy (IMRT) (five studies), Three-Dimensional Conformal Radiation Therapy (3D-CRT) (three studies), External Beam Radiation Therapy (EBRT) (three studies), and conventional radiotherapy (two studies). Note that three studies included two separate radiotherapy techniques. The standard treatment regimen observed across the studies typically spanned seven weeks, with radiotherapy administered five times per week. The predominant radiation dose identified in the studies was 2 Gy (range: 2–2.4 Gy) per day, reflecting standard fractionation protocols aimed at maximizing tumor control while minimizing toxicity to surrounding healthy tissues.

In the group of patients receiving weekly cisplatin, the difference between the mean planned dose of 68.49 Gy and the mean delivered dose of 67.16 Gy was not statistically significant (*p* = 0.16). Similarly, in the group receiving triweekly cisplatin, no significant difference was observed between the mean planned dose of 68.49 Gy and the mean delivered dose of 67.07 Gy (*p* = 0.14). Furthermore, the mean delivered radiation doses in the weekly (67.16 Gy) and triweekly (67.07 Gy) groups did not differ significantly (*p* = 0.48), indicating comparable adherence to the prescribed radiotherapy regimens in both treatment strategies.

### 3.4. Therapeutic Efficacy

A comparison of the treatment outcomes between the weekly and triweekly cisplatin regimens revealed no statistically significant differences across the evaluated parameters (Table 3). The complete response rate was slightly higher in the triweekly regimen, with a mean of 67.13%, compared to 63.18% in the weekly group (*p* = 0.32). The partial response rates were similar, averaging 30.34% for the weekly regimen and 28.12% for the triweekly regimen (*p* = 0.43).

Locoregional control at 2 years was higher in the triweekly regimen group, with a mean of 66.94% compared to 59.62% in the weekly group, although this difference did not reach statistical significance (*p* = 0.19).

**Figure 4:**
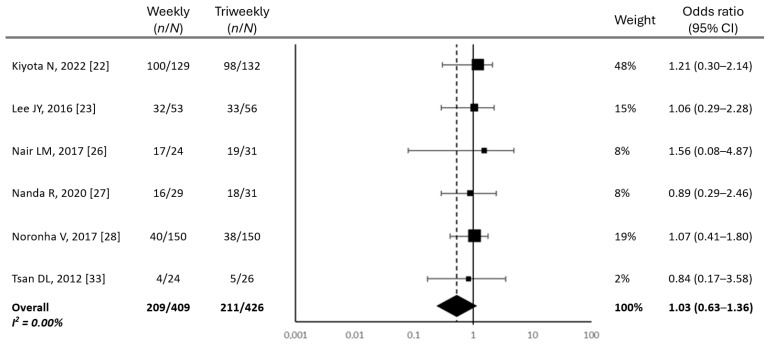
Forest plot of odds ratios for overall survival in weekly and triweekly cisplatin regimens. Abbreviations: CI, confidence interval; *n*, number of patients who completed chemotherapy; *N*, number of patients ([22,23,26,27,28,33]).

Overall survival at 2 years was comparable between the two regimens, with means of 51.24% for the weekly regimen and 49.47% for the triweekly regimen (*p* = 0.45) (Figure 4).

### 3.5. Treatment-Related Toxicity

The administration of cisplatin, whether in a weekly or triweekly regimen, is associated with a broad spectrum of adverse effects, which vary in severity and frequency. Due to the considerable heterogeneity in reported symptoms across the selected studies, the analysis was limited to adverse effects that were documented in at least four publications. All adverse events noted in the included publications are presented in the Supplementary Materials (Tables S2 and S3).

Separate analyses were conducted for acute adverse events of any grade (Table 4) and grade ≥ 3 toxicities, which represent severe or life-threatening complications (Table 5).

The incidences of overall acute toxicities of grade ≥ 3 were 56.95% in the weekly regimen and 67.05% in the triweekly regimen, with no statistically significant difference between the groups (*p* = 0.28). There were no statistically significant differences in the distributions of adverse events between the two regimens. These results suggests that the overall toxicity profile of cisplatin is broadly similar, regardless of the dosing frequency.

An analysis of the treatment-related mortality rates showed a slightly higher mean value in the triweekly regimen (2.92%) compared to the weekly regimen (2.29%), but the difference was not statistically significant (*p* = 0.35).

## 4. Discussion

To our knowledge, this is the first systematic review and meta-analysis of prospective clinical trials consisting of two arms: a low-dose 30–50 mg/m^2^ weekly regimen and a high-dose 100 mg/m^2^ triweekly regimen. An analysis was conducted to determine which of the two treatment regimens offers greater benefits for patients. The primary focus was to compare the treatment compliance, efficacy, and toxicity between the two regimens to identify which regimen might provide better outcomes in terms of both clinical effectiveness and tolerability.

The cumulative dose of cisplatin administered was significantly higher in the triweekly group compared to the weekly group, which was consistent with previous observations [28,35,36]. However, despite this difference, the proportion of patients who received a total dose of ≥200 mg/m^2^ did not differ significantly between the two regimens. A cumulative dose of ≥200 mg/m^2^ is widely considered the threshold required to achieve meaningful radiosensitization and improve locoregional control and overall survival in patients undergoing chemoradiotherapy for LA-HNSCC [19,37]. This cutoff was first proposed by Ang et al. [37], and subsequent clinical studies have confirmed its prognostic relevance. For instance, Al-Mamgani et al. [10] reported significantly better oncologic outcomes in patients who received at least 200 mg/m^2^ of cisplatin, a finding supported by Bhattacharjee et al. [11]. These observations suggest that in clinical practice both dosing schedules are capable of delivering a therapeutically adequate cumulative dose in the majority of patients, despite differences in the total dosage.

In regard to radiotherapy, in both the weekly and triweekly cisplatin groups, the planned and delivered doses did not differ significantly.

Statistical analysis found no significant differences in terms of efficacy between the two treatment groups. Although the triweekly regimen showed slightly higher complete response rates and better locoregional control, these differences did not reach statistical significance, suggesting that both regimens exhibit comparable efficacy in these aspects. The partial response rates were also similar between the two groups. Additionally, overall survival at 2 years was nearly identical across the two regimens, reinforcing the conclusion that the weekly and triweekly schedules do not differ significantly in terms of long-term outcomes. These findings suggest that while slight trends favoring the triweekly regimen were observed, the clinical relevance of these differences remains uncertain.

The results of the treatment-related toxicity analysis suggest that the overall toxicity profiles of the weekly and triweekly cisplatin regimens are broadly similar, with no statistically significant differences observed in the incidences of toxicities of any grade or grade ≥ 3. However, several important considerations must be taken into account when interpreting these findings. First, substantial heterogeneity was observed in the reported rates of toxicity across the studies, with wide ranges documented for several adverse effects (e.g., thrombocytopenia of any grade in the weekly regimen: 2.70–100.00%). Several factors may explain this wide variation. First, although the majority of the included trials employed the Common Terminology Criteria for Adverse Events (CTCAE) version 4.0, a subset of the studies used CTCAE version 3.0, the Radiation Therapy Oncology Group (RTOG) Acute Radiation Morbidity Criteria, the WHO toxicity criteria, or the RTOG/EORTC late toxicity scales. Moreover, some studies did not specify the grading system used. These differences likely introduced systematic inconsistencies in the toxicity reporting, especially for subjectively assessed endpoints such as mucositis, nausea, and dysphagia, and may therefore account for the broad range of reported toxicity rates observed across the studies.

Second, differences in institutional supportive care practices, including the use of prophylactic oral rinses, analgesia protocols, and nutritional support, may have mitigated or exacerbated the manifestation of toxicities, thereby influencing their frequency and severity. Third, variability in patient-related characteristics, i.e., age, comorbidities, and nutritional status, also could have contributed to inter-study differences in toxicity profiles. Lastly, discrepancies in data collection methods, including the timing of toxicity assessment and whether peak or cumulative grades were reported, further complicated direct comparisons between studies. These factors collectively underscore the importance of methodological standardization in toxicity assessment and reporting, which would significantly enhance the interpretability of pooled data in future meta-analyses. Although the selected studies were chosen to ensure maximal similarity in their designs and treatment protocols, subtle, unaccounted-for differences in these aspects may have contributed to the heterogeneity in the reported toxicity rates.

Furthermore, the variability in data reporting across the included studies led to certain outcomes being reported in only one or two publications. The small number of studies reporting specific toxicities limited the statistical power to detect significant differences, particularly for rare but clinically relevant severe adverse events. In consequence, it was not possible to draw reliable conclusions, as the sample size was too small to provide sufficient evidence for generalization. Additionally, while the *p*-values did not reach the threshold for statistical significance, trends in some toxicities, such as higher rates of grade ≥ 3 nausea/vomiting and thrombocytopenia in the triweekly regimen, may carry clinical relevance and require further investigation. The high overall rates of toxicities underscore the importance of individualized patient management, including proactive supportive care to mitigate treatment-related complications. The difference in treatment-related mortality between the two compared regimens was not statistically significant.

One of the factors complicating a clear comparison of the treatment compliance in the two regimens was the transparency of the reports of treatment discontinuation or delays, which could have affected the cumulative cisplatin doses. Another challenge was the variability in how the study authors defined the completion of the planned treatments. In some publications, this was interpreted as completing all planned cycles, while in others, it referred to completing a specific number of cycles, which was not always equivalent to the total number of planned cycles. Incomplete or inconsistent reporting of these factors made it difficult to accurately assess the true dose intensity and adherence to the prescribed regimen.

The pooled results of this meta-analysis suggest that the weekly and triweekly cisplatin regimens have broadly comparable efficacy and toxicity profiles. However, clinical heterogeneity among the included patient populations may have influenced the observed outcomes. Although we only included studies focusing on LA-HNSCC treated with cisplatin-based chemoradiotherapy, the trials varied in terms of tumor stage distribution, baseline performance status, comorbidities, postoperative versus definitive treatment settings, and other factors that may affect treatment tolerance and effectiveness. Due to limitations in the available data, subgroup-level analyses could not be thoroughly performed. Future prospective, randomized trials are warranted to directly compare these regimens in well-defined clinical subgroups, such as elderly or frail patients, those receiving postoperative versus definitive chemoradiotherapy, or individuals with site-specific disease characteristics. Stratification by tumor site and detailed toxicity phenotyping may help to tailor regimen selection to individual patient needs. Until such data become available, the choice between weekly and triweekly cisplatin should be guided by patient-specific clinical factors, rather than an assumed overall superiority of one schedule.

In addition to subgroup-related considerations, several other methodological limitations should be acknowledged. Although our search strategy was comprehensive and included multiple databases, the possibility of publication bias cannot be entirely excluded. To mitigate bias and improve the reliability of our analysis, we focused exclusively on prospective trials. This approach ensured a higher level of methodological rigor; however, it significantly limited the number of eligible studies, which may have impacted the statistical significance of the results. Studies with inconclusive or nonsignificant findings may be underreported or less likely to be indexed, potentially skewing the available evidence base. However, a formal statistical assessment of publication bias (e.g., funnel plot asymmetry and Egger’s test) could not be performed due to the limited number of studies contributing to each specific outcome.

A risk-of-bias assessment was performed using the Cochrane RoB 2.0 tool, and detailed evaluations are presented in the Supplementary Materials. Several trials were rated as having “some concerns” or a “high risk of bias”, primarily due to a lack of blinding, as most included studies were conducted as open-label trials. In cases where the blinding status was not explicitly stated, it was conservatively assumed to be absent. This methodological limitation significantly affected the overall RoB judgments, as the tool places substantial emphasis on blinding in domains such as adherence to interventions and outcome assessment. Nevertheless, it is important to emphasize that the primary endpoints of our meta-analysis, overall survival and chemotherapy completion, are objective measures that are less susceptible to observer and performance bias. Therefore, while the formal RoB assessments indicate moderate-to-high risk, we believe their practical impact on the pooled outcomes is limited.

A sensitivity analysis excluding high-risk studies was considered; however, the limited number of trials would have significantly reduced the statistical power and generalizability of the findings. We acknowledge this as a limitation and recommend that future trials ensure clear and complete reporting of methodological features relevant to bias assessment, including blinding and protocol deviations.

Second, due to variability in outcome reporting and missing data across the studies, our analyses were limited to parameters reported by at least four publications. No imputation was performed for missing data, which may have reduced the statistical power for certain toxicity endpoints and may limit the generalizability of those findings.

Future research should go beyond efficacy and toxicity endpoints and incorporate health economics, particularly cost-effectiveness analyses of weekly versus triweekly regimens, which may differ substantially in resource utilization (e.g., infusion visits, supportive care, and monitoring). Furthermore, the identification of predictive biomarkers, including genomic, proteomic, or imaging-derived markers, could support personalized regimen selection and improve outcomes.

Finally, while we extracted the available information on the radiotherapy techniques used in the included studies, it was not possible to systematically evaluate the impacts of specific modalities (e.g., IMRT vs. 3D-CRT) on treatment outcomes. This limitation stemmed from two main factors: first, the inconsistent or incomplete reporting of radiotherapy techniques across the studies and second, the fact that this meta-analysis was based on aggregate data, which precluded correlating patient-level outcomes with treatment parameters such as the radiation modality. Future individual patient data (IPD) meta-analyses or prospective trials with predefined stratification by radiotherapy technique are needed to clarify the potential influence of the radiation delivery method on efficacy and toxicity.

In the meta-analysis conducted by Szturz et al. [38], the assessment of treatment efficacy and grade ≥ 3 toxicities was stratified based on definitive and postoperative treatment settings. Their findings indicate that in the postoperative setting, dysphagia and significant weight loss occurred significantly less frequently in patients receiving the triweekly regimen compared to those treated with the weekly regimen. However, for other widely reported adverse effects, no statistically significant differences were observed between the two regimens. In contrast, in the definitive setting, severe leukopenia, neutropenia, nausea/vomiting, and renal toxicity were significantly less common in patients receiving the weekly regimen compared to those treated with the triweekly regimen. Unlike the meta-analysis by Szturz et al. [38], which included single-arm studies, our analysis only accepted studies with two study arms. Despite these differences, both the meta-analysis by Szturz et al. [38] and our analysis did not conclusively identify which treatment regimen is superior.

## 5. Conclusions

The findings suggest that both cisplatin regimens remain viable treatment options, as they achieve comparable efficacy, with no significant differences in overall survival, locoregional control, or complete response rates. While the triweekly regimen resulted in a higher cumulative cisplatin dose, this did not translate into significantly improved treatment outcomes. Treatment adherence was similar in both regimens, suggesting that despite concerns, poor compliance with high-dose cisplatin may not be as clinically impactful as previously thought. In terms of toxicity, no statistically significant differences were observed in overall or severe (grade ≥ 3) adverse effects, and the mortality rates did not differ between the two regimens.

The choice of regimen should be guided by patient-specific factors, including toxicity risk, treatment tolerability, and institutional protocols. Future studies should focus on standardizing toxicity assessments, evaluating long-term treatment outcomes, and identifying patient subgroups that may benefit preferentially from one regimen over the other.

## Figures and Tables

**Table 1:** A summary of the studies meeting the inclusion criteria. Abbreviations: Q1, weekly; Q3, every three weeks.

Author, Year	Study Type/Phase	Randomized	Recruitment Dates	Country	Tumor Resection Status	Study Arms	Number of Patients (*n*)
Ameri A, 2021 [12]	Prospective	Yes	-	Iran	Mostly unresected	40 mg/m^2^ Q1	39
						100 mg/m^2^ Q3	38
Chaturvedi A, 2024 [13]	Prospective	Yes	May 2016–June 2019	India	Unresected	35 mg/m^2^ Q1	26
						100 mg/m^2^ Q3	25
Kiyota N, 2022 [22]	Phase II/III	Yes	October 2012–December 2018	Japan	Resected	40 mg/m^2^ Q1	129
						100 mg/m^2^ Q3	132
Lee JY, 2016 [23]	Phase II	Yes	September 2009–December 2013	South Korea	-	40 mg/m^2^ Q1	53
						100 mg/m^2^ Q3	56
Mashhour K, 2020 [24]	Prospective	Yes	July 2016–July 2019	Egypt	Both	30 mg/m^2^ Q1	30
						100 mg/m^2^ Q3	30
Mitra D, 2011 [25]	Prospective	Yes	February 2010–January 2011	India	Unresected	30 mg/m^2^ Q1	30
						100 mg/m^2^ Q3	30
Nair LM, 2017 [26]	Phase IIb	Yes	June 2013–May 2014	India	Unresected	40 mg/m^2^ Q1	24
						100 mg/m^2^ Q3	31
Nanda R, 2020 [27]	Prospective	Yes	December 2010–January 2013	India	Unresected	40 mg/m^2^ Q1	29
						100 mg/m^2^ Q3	31
Noronha V, 2017 [28]	Phase III	Yes	2013–2017	India	Mostly resected	30 mg/m^2^ Q1	150
						100 mg/m^2^ Q3	150
Panihar C, 2022 [29]	Prospective	Yes	December 2017–May 2019	India	Unresected	40 mg/m^2^ Q1	44
						100 mg/m^2^ Q3	41
Rawat S, 2016 [30]	Prospective	Yes	June 2013–March 2014	India	Unresected	35 mg/m^2^ Q1	30
						100 mg/m^2^ Q3	30
Sahoo TK, 2017 [31]	Prospective	Yes	November 2011–October 2012	India	Unresected	30 mg/m^2^ Q1	15
						100 mg/m^2^ Q3	15
Sharma A, 2022 [32]	Phase III	-	April 2018–January 2021	India	Unresected	40 mg/m^2^ Q1	132
						100 mg/m^2^ Q3	132
Tsan DL, 2012 [33]	Phase III	Yes	February 2008–August 2010	Taiwan	Resected	40 mg/m^2^ Q1	24
						100 mg/m^2^ Q3	26
Uygun K, 2009 [34]	Prospective	-	January 2002–December 2007	Turkey	Unresected	40 mg/m^2^ Q1	20
						100 mg/m^2^ Q3	30

**Table 2:** Treatment compliance. Number of studies refers to studies that reported given characteristic. Bolded *p*-values indicate those of statistical significance. Abbreviations: CI, confidence interval; *n*, number.

Characteristic	Number of Studies [*n*]	Mean Value (95% CI)		*p*-Value
		Weekly	Triweekly	
Chemotherapy completion [%]	12	74.76% (65.35–84.16)	72.29% (59.42–85.15)	0.38
Cumulative cisplatin dose [mg/m^2^]	11	241.74 (213.94–269.55)	287.52 (247.75–327.30)	**0.04**
Cumulative cisplatin dose ≥200 mg/m^2^ [%]	7	70.64% (57.30–83.98)	78.75% (63.36–94.14)	0.23

**Table 3:** Treatment outcomes. Number of studies refers to studies that reported given characteristic. Abbreviation: CI, confidence interval.

Characteristic	Number of Studies [*n*]	Mean Value (95% CI) [%]		*p*-Value
		Weekly	Triweekly	
Complete response [%]	11	63.18% (51.72–74.63)	67.13% (55.50–78.76)	0.32
Partial response [%]	7	30.34% (14.34–46.34)	28.12% (11.66–44.57)	0.43
Locoregional control at 2 years [%]	7	59.62% (49.36–69.89)	66.94% (55.37–78.50)	0.19
Overall survival at 2 years [%]	6	51.24% (31.68–70.79)	49.47% (31.91–67.02)	0.45

**Table 4:** Incidence of acute toxicity of any grade.

Toxicity	Number of Studies [*n*]	Median (Min–Max) [%]		*p*-Value
		Weekly	Triweekly	
Hematological				
Anemia	11	73.37% (8.30–100.00)	76.6% (22.50–100.00)	0.34
Leukopenia	7	86.67% (20.27–100.00)	95.00% (46.98–100.00)	0.20
Neutropenia	10	66.42% (9.46–100.00)	81.00% (30.87–100.00)	0.19
Thrombocytopenia	10	46.50% (2.70–100.00)	53.50% (2.70–100.00)	0.44
Non-hematological				
Dermatitis	9	100.00% (61.49–100.00)	100.00% (62.42–100.00)	0.46
Dysphagia	7	95.83% (48.00–100.00)	100.00% (58.00–100.00)	0.44
Mucositis	9	100.00% (81.76–100.00)	100.00% (90.60–100.00)	0.35
Nausea/vomiting	9	93.37% (15.54–100.00)	100.00% (29.53–100.00)	0.33
Renal toxicity	4	3.75% (0.00–16.67)	5.26% (0.00–9.68)	0.41
Significant weight loss (>10%)	4	27.15% (11.49–43.60)	40.70% (17.45–42.10)	0.21

**Table 5:** The incidence of grade ≥ 3 acute toxicity. * Grade ≥ 3 anemia in the triweekly regimen was not assessed in one of the publications that reported anemia in the weekly regimen.

Toxicity	Number of Studies [*n*]	Median (Min–Max) [%]		*p*-Value
		Weekly	Triweekly	
Overall acute toxicities of grade ≥ 3	4	56.95% (40.00–71.62)	67.05% (39.30–84.56)	0.28
Hematological				
Anemia	11/10 *	4.20% (0.00–26.70)	6.64% (0.00–36.70)	0.23
Leukopenia	7	20.00% (2.70–62.00)	16.11% (0.00–55.00)	0.46
Neutropenia	10	16.45% (1.35–35.00)	18.05% (0.00–49.00)	0.30
Thrombocytopenia	10	2.85% (0.00–7.50)	2.66% (0.00–16.60)	0.14
Non-hematological				
Dermatitis	10	13.79% (6.76–38.00)	11.65% (3.20–64.00)	0.48
Dysphagia	8	44.70% (0.00–85.00)	35.61% (6.67–92.00)	0.50
Mucositis	11	53.40% (15.54–85.00)	46.70% (18.12–92.00)	0.50
Nausea/vomiting	9	7.50% (1.35–20.80)	13.00% (0.00–40.00)	0.11
Renal toxicity	6	0.00% (0.00–5.00)	0.00% (0.00–16.60)	0.26

## Data Availability

All data necessary to reproduce the analyses presented in this study will be made available upon request to the corresponding author. The code used for this manuscript is available at https://github.com/PBS-Bydgoszcz/cisplatin_regimen_comparison.git, accessed on 2 March 2025.

## Supplementary Materials

The following supporting information can be downloaded at https://www.mdpi.com/article/10.3390/cancers17091444/s1, Table S1: Patients characteristics in the studies meeting inclusion criteria; Table S2: Acute adverse events in the studies meeting inclusion criteria. Abbreviations: n, number of patients assessed for safety; NR, not reported; Table S3: Long term adverse events in the studies meeting inclusion criteria. Studies that did not report any long term adverse events are not presented. Abbreviations: n, number of patients assessed for safety; NR, not reported.
