# Supplementary material for: Comparison of Weekly and Triweekly Cisplatin Regimens in the Treatment of Head and Neck Cancer: A Systematic Review and Meta-Analysis
Source: Cancers (Basel). 2025 Apr 25;17(9):1444. doi: 10.3390/cancers17091444 (PMC12071001; doi:10.3390/cancers17091444)
Supplement: Supplementary file 1 [file cancers-17-01444-s001.zip › Supplementary data.pdf]

Comparison of Weekly and Triweekly Cisplatin Regimens in the Treatment of Head and Neck Cancer: A Systematic Review and Meta-Analysis

Sylwester M. Kloska * and Anna Kloska

Faculty of Medicine, Bydgoszcz University of Science and Technology, 85-796 Bydgoszcz, Poland; anna.kloska@pbs.edu.pl
* Correspondence: sylwester.kloska@pbs.edu.pl

Table S1 Patients characteristics in the studies meeting inclusion criteria.

Author, year	Study arm	Number of patients [n]	Sex [%]		Age [years]	Disease stage [%]					Primary tumor site [%]		
			Male	Female		II	III	IV	IVA	IVB	Nasopharynx, nasal cavity, paranasal-sinuses	Oropharynx, oral cavity, salivary glands	Larynx, hypopharynx
Ameri A, 2021	Weekly	39	79.5	20.5	55.5	5.1	23.1	71.8	NR	NR	15.4	28.2	56.4
	Triweekly	38	76.3	23.7	55.2	0.0	21.1	78.9	NR	NR	18.4	26.3	55.3
Chaturvedi A, 2024	Weekly	26	88.0	12.0	NR	0.0	35.0	NR	50.0	15.0	0.0	80.8	19.2
	Triweekly	25	92.0	8.0	NR	0.0	16.0	NR	64.0	20.0	0.0	68.0	32.0
Kiyota N, 2022	Weekly	129	85.3	14.7	61.0	0.0	9.0	NR	88.0	2.0	0.0	62.8	37.2
	Triweekly	132	83.3	16.7	62.0	0.0	7.0	NR	88.0	4.0	0.0	56.8	43.2
Lee JY, 2016	Weekly	53	73.6	26.4	53.6	28.3	45.3	26.4	NR	NR	NR	NR	NR
	Triweekly	56	83.9	16.1	52.7	17.9	57.1	25.0	NR	NR	NR	NR	NR
Mashhour K, 2020	Weekly	30	73.3	26.7	60.0	0.0	33.3	NR	36.6	30.1	13.3	36.7	50.0
	Triweekly	30	80.0	20.0	61.0	0.0	30.1	NR	36.6	33.1	23.3	36.7	40.0
Mitra D, 2011	Weekly	30	93.3	6.7	51.0	0.0	53.3	46.7	NR	NR	6.7	33.3	60.0
	Triweekly	30	90.0	10.0	52.0	0.0	56.7	43.3	NR	NR	10.0	36.7	53.3
Nair LM, 2017	Weekly	24	95.8	4.2	NR	0.0	62.5	37.5	NR	NR	0.0	41.7	58.3
	Triweekly	31	90.3	9.7	NR	0.0	58.1	41.9	NR	NR	0.0	54.8	45.2
Nanda R, 2020	Weekly	29	93.1	6.9	54.4	0.0	34.5	65.5	NR	NR	0.0	100.0	0.0
	Triweekly	31	96.8	3.2	55.0	0.0	35.5	64.5	NR	NR	0.0	100.0	0.0
Noronha V, 2017	Weekly	150	90.0	10.0	41.5	0.0	8.7	91.3	NR	NR	0.0	92.0	3.3
	Triweekly	150	88.0	12.0	47.0	0.0	8.7	91.3	NR	NR	0.0	88.0	7.3
Panihar C, 2022	Weekly	44	88.6	11.4	54.9	0.0	45.5	NR	54.5	NR	0.0	68.2	31.8
	Triweekly	41	82.9	17.1	53.9	0.0	36.6	NR	63.4	NR	0.0	65.9	34.1
Rawat S, 2016	Weekly	30	90.0	10.0	50.7	0.0	23.3	76.7	NR	NR	0.0	80.0	20.0
	Triweekly	30	100.0	0.0	51.4	0.0	40.0	60.0	NR	NR	0.0	76.7	23.3
Sahoo TK, 2017	Weekly	15	NR	NR	NR	NR	NR	NR	NR	NR	NR	NR	NR
	Triweekly	15	NR	NR	NR	NR	NR	NR	NR	NR	NR	NR	NR
Sharma A, 2022	Weekly	132	NR	NR	NR	NR	NR	NR	NR	NR	NR	NR	NR
	Triweekly	132	NR	NR	NR	NR	NR	NR	NR	NR	NR	NR	NR

Tsan DL, 2012	Weekly<br>Triweekly	24<br>26	95.8<br>96.2	4.2<br>3.8	49.0<br>49.2	4.2<br>0.0	16.7<br>11.5	79.2<br>88.5	NR<br>NR	NR<br>NR	0.0<br>0.0	100.0<br>100.0	0.0<br>0.0
Uygun K, 2009	Weekly<br>Triweekly	20<br>30	75.0<br>80.0	25.0<br>20.0	71.0<br>53.2	0.0<br>0.0	20.0<br>30.0	80.0<br>70.0	NR<br>NR	NR<br>NR	0.0<br>0.0	10.0<br>10.0	90.0<br>90.0

Table S2 Acute adverse events in the studies meeting inclusion criteria. Abbreviations: n, number of patients assessed for safety; NR, not reported.

	Ameri A, 2021		Chaturvedi A, 2024		Kiyota N, 2022		Lee JY, 2016		Mashhour K, 2020		Mitra D, 2011		Nair LM, 2017		Nanda R, 2020		Noronha V, 2017		Panihar C, 2022		Rawat S, 2016		Sahoo TK, 2017		Sharma A, 2022		Tsan DL, 2012		Uygun K, 2009	
Toxicity	Weekly (n=39)	Triweekly (n=38)	Weekly (n=26)	Triweekly (n=25)	Weekly (n=122)	Triweekly (n=129)	Weekly (n=53)	Triweekly (n=56)	Weekly (n=30)	Triweekly (n=30)	Weekly (n=30)	Triweekly (n=30)	Weekly (n=25)	Triweekly (n=31)	Weekly (n=29)	Triweekly (n=31)	Weekly (n=148)	Triweekly (n=149)	Weekly (n=44)	Triweekly (n=41)	Weekly (n=30)	Triweekly (n=29)	Weekly (n=15)	Triweekly (n=15)	Weekly (n=132)	Triweekly (n=132)	Weekly (n=24)	Triweekly (n=26)	Weekly (n=20)	Triweekly (n=30)
Any grade ≥3 toxicity (%)	NR	NR	NR	NR	NR	NR	47.20	39.30	NR	NR	NR	NR	NR	NR	NR	NR	71.62	84.56	NR	NR	NR	NR	NR	NR	NR	NR	66.70	80.80	40.00	53.30
Non-hematological toxicities grade ≥3 (%)	NR	NR	NR	NR	NR	NR	NR	NR	56.60	76.60	NR	NR	NR	NR	NR	NR	NR	NR	NR	NR	NR	NR	NR	NR	NR	NR	NR	NR	NR	NR
Alopecia any grade (%)	NR	NR	NR	NR	12.00	18.00	NR	NR	NR	NR	NR	NR	NR	NR	NR	NR	NR	NR	NR	NR	NR	NR	NR	NR	NR	NR	NR	NR	NR	NR
ALT increased any grade (%)	NR	NR	NR	NR	30.00	57.00	NR	NR	NR	NR	NR	NR	NR	NR	NR	NR	NR	NR	NR	NR	NR	NR	NR	NR	NR	NR	NR	NR	NR	NR
ALT increased grade ≥3 (%)	NR	NR	NR	NR	3.00	3.00	NR	NR	NR	NR	NR	NR	NR	NR	NR	NR	NR	NR	NR	NR	NR	NR	NR	NR	NR	NR	NR	NR	NR	NR
Anemia any grade (%)	NR	NR	31.00	48.00	100.00	100.00	100.00	100.00	50.00	76.60	100.00	100.00	8.30	22.50	100.00	100.00	14.19	32.21	NR	NR	33.30	31.00	73.37	66.67	NR	NR	100.00	100.00	NR	NR
Anemia grade ≥3 (%)	2.60	NR	NR	NR	13.00	14.00	1.90	8.90	16.70	29.90	26.70	36.70	4.10	0.00	0.00	3.30	2.03	4.70	NR	NR	NR	NR	6.67	6.67	NR	NR	4.20	3.80	10.00	6.60
AST increased any grade (%)	NR	NR	NR	NR	30.00	51.00	NR	NR	NR	NR	NR	NR	NR	NR	NR	NR	NR	NR	NR	NR	NR	NR	NR	NR	NR	NR	NR	NR	NR	NR
AST increased grade ≥3 (%)	NR	NR	NR	NR	0.00	3.00	NR	NR	NR	NR	NR	NR	NR	NR	NR	NR	NR	NR	NR	NR	NR	NR	NR	NR	NR	NR	NR	NR	NR	NR
Constipation any grade (%)	NR	NR	NR	NR	46.00	49.00	NR	NR	NR	NR	NR	NR	NR	NR	NR	NR	NR	NR	NR	NR	NR	NR	NR	NR	NR	NR	NR	NR	NR	NR
Constipation grade ≥3 (%)	NR	NR	NR	NR	0.00	0.00	NR	NR	NR	NR	NR	NR	NR	NR	NR	NR	NR	NR	NR	NR	NR	NR	NR	NR	NR	NR	NR	NR	NR	NR
Creatinine increased any grade (%)	NR	NR	NR	NR	30.00	40.00	NR	NR	NR	NR	NR	NR	NR	NR	NR	NR	NR	NR	NR	NR	NR	NR	NR	NR	NR	NR	NR	NR	NR	NR
Creatinine increased grade ≥3 (%)	NR	NR	NR	NR	0.00	0.00	NR	NR	NR	NR	NR	NR	NR	NR	NR	NR	NR	NR	NR	NR	NR	NR	NR	NR	NR	NR	NR	NR	NR	NR
Dermatitis any grade (%)	NR	NR	100.00	100.00	92.00	92.00	NR	NR	100.00	100.00	100.00	100.00	100.00	100.00	100.00	100.00	61.49	62.42	NR	NR	NR	NR	100.00	93.33	NR	NR	100.00	100.00	NR	NR
Dermatitis grade ≥3 (%)	NR	NR	38.00	64.00	12.00	15.00	NR	NR	13.30	10.00	16.67	13.33	12.50	3.20	14.28	19.35	6.76	8.05	NR	NR	NR	NR	26.67	6.67	NR	NR	8.30	7.70	15.00	13.30
Diarrhea any grade (%)	NR	NR	NR	NR	12.00	20.00	NR	NR	NR	NR	NR	NR	NR	NR	NR	NR	8.78	14.77	NR	NR	NR	NR	NR	NR	NR	NR	NR	NR	NR	NR
Diarrhea grade ≥3 (%)	NR	NR	NR	NR	1.00	0.00	NR	NR	NR	NR	NR	NR	NR	NR	NR	NR	1.35	5.37	NR	NR	NR	NR	NR	NR	NR	NR	NR	NR	NR	NR
Dysgeusia any grade (%)	NR	NR	NR	NR	66.00	75.00	NR	NR	NR	NR	NR	NR	NR	NR	NR	NR	51.35	63.09	NR	NR	NR	NR	NR	NR	NR	NR	NR	NR	NR	NR
Dysgeusia grade ≥3 (%)	NR	NR	NR	NR	NR	NR	NR	NR	NR	NR	NR	NR	NR	NR	NR	NR	0.00	0.67	NR	NR	NR	NR	NR	NR	NR	NR	NR	NR	NR	NR
Dysphagia any grade (%)	NR	NR	100.00	100.00	48.00	58.00	NR	NR	100.00	100.00	NR	NR	95.83	93.50	100.00	100.00	78.38	73.83	NR	NR	NR	NR	93.33	100.00	NR	NR	NR	NR	NR	NR
Dysphagia grade ≥3 (%)	NR	NR	85.00	92.00	12.00	19.00	NR	NR	46.70	66.70	NR	NR	62.50	25.80	46.40	32.30	42.57	38.93	43.00	56.00	NR	NR	0.00	6.67	NR	NR	NR	NR	NR	NR

Fatigue any grade (%)	NR	NR	NR	NR	34.00	39.00	NR	NR	NR	NR	NR	NR	NR	NR	NR	NR	12.84	14.09	NR	NR	NR	NR	NR	NR	NR	NR	NR	NR	NR	NR
Fatigue grade ≥3 (%)	NR	NR	NR	NR	1.00	4.00	NR	NR	NR	NR	NR	NR	NR	NR	NR	NR	0.68	0.67	NR	NR	NR	NR	NR	NR	NR	NR	NR	NR	NR	NR
Febrile neutropenia any grade (%)	NR	NR	NR	NR	5.00	5.00	NR	NR	NR	NR	NR	NR	NR	NR	NR	NR	NR	NR	NR	NR	NR	NR	NR	NR	NR	NR	NR	NR	NR	NR
Febrile neutropenia grade ≥3 (%)	NR	NR	NR	NR	5.00	5.00	NR	NR	NR	NR	NR	NR	NR	NR	NR	NR	0.68	6.04	NR	NR	NR	NR	NR	NR	NR	NR	NR	NR	NR	NR
Hearing disturbance any grade (%)	NR	NR	NR	NR	7.00	17.00	NR	NR	NR	NR	NR	NR	NR	NR	NR	NR	NR	NR	NR	NR	NR	NR	NR	NR	NR	NR	NR	NR	NR	NR
Hearing disturbance grade ≥3 (%)	NR	NR	NR	NR	2.00	4.00	NR	NR	NR	NR	NR	NR	NR	NR	NR	NR	NR	NR	NR	NR	NR	NR	NR	NR	NR	NR	NR	NR	NR	NR
Hearing loss any grade (%)	NR	NR	NR	NR	NR	NR	1.89	1.79	NR	NR	NR	NR	NR	NR	NR	NR	8.11	19.46	NR	NR	NR	NR	NR	NR	NR	NR	NR	NR	NR	NR
Hearing loss grade ≥3 (%)	NR	NR	NR	NR	NR	NR	NR	NR	NR	NR	NR	NR	NR	NR	NR	NR	4.73	12.75	NR	NR	NR	NR	NR	NR	NR	NR	NR	NR	NR	NR
Hematological toxicities grade ≥3 (%)	56.40	65.80	NR	NR	NR	NR	NR	NR	NR	NR	NR	NR	NR	NR	NR	NR	NR	NR	NR	NR	NR	NR	NR	NR	NR	NR	NR	NR	NR	NR
Hoarseness any grade (%)	NR	NR	NR	NR	NR	NR	NR	NR	NR	NR	NR	NR	NR	NR	NR	NR	45.95	51.68	NR	NR	NR	NR	NR	NR	NR	NR	NR	NR	NR	NR
Hoarseness grade ≥3 (%)	NR	NR	NR	NR	NR	NR	NR	NR	NR	NR	NR	NR	NR	NR	NR	NR	8.78	9.40	NR	NR	NR	NR	NR	NR	NR	NR	NR	NR	NR	NR
Hypercalcemia any grade (%)	NR	NR	NR	NR	12.00	13.00	NR	NR	NR	NR	NR	NR	NR	NR	NR	NR	NR	NR	NR	NR	NR	NR	NR	NR	NR	NR	NR	NR	NR	NR
Hypercalcemia grade ≥3 (%)	NR	NR	NR	NR	1.00	1.00	NR	NR	NR	NR	NR	NR	NR	NR	NR	NR	NR	NR	NR	NR	NR	NR	NR	NR	NR	NR	NR	NR	NR	NR
Hyperkalemia any grade (%)	NR	NR	NR	NR	63.00	61.00	NR	NR	NR	NR	NR	NR	NR	NR	NR	NR	NR	NR	NR	NR	NR	NR	NR	NR	NR	NR	NR	NR	NR	NR
Hyperkalemia grade ≥3 (%)	NR	NR	NR	NR	2.00	5.00	NR	NR	NR	NR	NR	NR	NR	NR	NR	NR	1.35	5.37	NR	NR	NR	NR	NR	NR	NR	NR	NR	NR	NR	NR
Hypermagnesemia any grade (%)	NR	NR	NR	NR	8.00	20.00	NR	NR	NR	NR	NR	NR	NR	NR	NR	NR	NR	NR	NR	NR	NR	NR	NR	NR	NR	NR	NR	NR	NR	NR
Hypermagnesemia grade ≥3 (%)	NR	NR	NR	NR	1.00	2.00	NR	NR	NR	NR	NR	NR	NR	NR	NR	NR	NR	NR	NR	NR	NR	NR	NR	NR	NR	NR	NR	NR	NR	NR
Hypertension any grade (%)	NR	NR	NR	NR	NR	NR	NR	NR	NR	NR	NR	NR	NR	NR	NR	NR	27.03	38.26	NR	NR	NR	NR	NR	NR	NR	NR	NR	NR	NR	NR
Hypertension grade ≥3 (%)	NR	NR	NR	NR	NR	NR	NR	NR	NR	NR	NR	NR	NR	NR	NR	NR	4.05	7.38	NR	NR	NR	NR	NR	NR	NR	NR	NR	NR	NR	NR
Hypoalbuminemia any grade (%)	NR	NR	NR	NR	96.00	100.00	NR	NR	NR	NR	NR	NR	NR	NR	NR	NR	NR	NR	NR	NR	NR	NR	NR	NR	NR	NR	NR	NR	NR	NR
Hypoalbuminemia grade ≥3 (%)	NR	NR	NR	NR	0.00	0.00	NR	NR	NR	NR	NR	NR	NR	NR	NR	NR	NR	NR	NR	NR	NR	NR	NR	NR	NR	NR	NR	NR	NR	NR
Hypocalcemia any grade (%)	NR	NR	NR	NR	55.00	62.00	NR	NR	NR	NR	NR	NR	NR	NR	NR	NR	NR	NR	NR	NR	NR	NR	NR	NR	NR	NR	NR	NR	NR	NR
Hypocalcemia grade ≥3 (%)	NR	NR	NR	NR	6.00	4.00	NR	NR	NR	NR	NR	NR	NR	NR	NR	NR	NR	NR	NR	NR	NR	NR	NR	NR	NR	NR	NR	NR	NR	NR
Hypokalemia any grade (%)	NR	NR	NR	NR	21.00	36.00	NR	NR	NR	NR	NR	NR	NR	NR	NR	NR	1.35	5.37	NR	NR	NR	NR	NR	NR	NR	NR	NR	NR	NR	NR
Hypokalemia grade ≥3 (%)	NR	NR	NR	NR	3.00	5.00	NR	NR	NR	NR	NR	NR	NR	NR	NR	NR	1.35	5.37	NR	NR	NR	NR	NR	NR	NR	NR	NR	NR	NR	NR
Hypomagnesemia any grade (%)	NR	NR	NR	NR	54.00	51.00	NR	NR	NR	NR	NR	NR	NR	NR	NR	NR	NR	NR	NR	NR	20.00	60.00	NR	NR	NR	NR	NR	NR	NR	NR
Hypomagnesemia grade ≥3 (%)	NR	NR	NR	NR	1.00	0.00	NR	NR	NR	NR	NR	NR	NR	NR	NR	NR	NR	NR	NR	NR	NR	NR	NR	NR	NR	NR	NR	NR	NR	NR
Hyponatremia any grade (%)	NR	NR	NR	NR	82.00	92.00	NR	NR	NR	NR	NR	NR	NR	NR	NR	NR	NR	NR	NR	NR	33.30	40.00	NR	NR	NR	NR	NR	NR	NR	NR
Hyponatremia grade ≥3 (%)	NR	NR	NR	NR	11.00	10.00	NR	NR	NR	NR	NR	NR	NR	NR	NR	NR	22.97	52.35	NR	NR	NR	NR	NR	NR	NR	NR	NR	NR	NR	NR
Infection any grade (%)	NR	NR	NR	NR	15.00	19.00	NR	NR	NR	NR	NR	NR	NR	NR	NR	NR	47.30	66.44	NR	NR	NR	NR	NR	NR	NR	NR	NR	NR	NR	NR

Infection grade ≥3 (%)	NR	NR	NR	NR	7.00	12.00	NR	NR	NR	NR	NR	NR	NR	NR	NR	NR	21.62	33.56	NR	NR	NR	NR	NR	NR	NR	NR	NR	NR	NR	NR
Laryngeal edema any grade (%)	NR	NR	NR	NR	3.00	6.00	NR	NR	100.00	100.00	NR	NR	NR	NR	NR	NR	NR	NR	NR	NR	NR	NR	NR	NR	NR	NR	100.00	100.00	NR	NR
Laryngeal edema grade ≥3 (%)	NR	NR	NR	NR	0.00	0.00	NR	NR	16.70	16.67	NR	NR	NR	NR	NR	NR	NR	NR	NR	NR	NR	NR	NR	NR	NR	NR	4.20	11.50	NR	NR
Larynx any grade (%)	NR	NR	NR	NR	NR	NR	NR	NR	NR	NR	NR	NR	NR	NR	100.00	100.00	NR	NR	NR	NR	NR	NR	NR	NR	NR	NR	NR	NR	NR	NR
Larynx grade ≥3 (%)	NR	NR	NR	NR	NR	NR	NR	NR	NR	NR	NR	NR	NR	NR	10.70	9.70	NR	NR	NR	NR	NR	NR	NR	NR	NR	NR	NR	NR	NR	NR
Leukopenia any grade (%)	NR	NR	NR	NR	93.00	95.00	NR	NR	60.00	96.60	NR	NR	NR	NR	100.00	100.00	20.27	46.98	64.00	93.00	NR	NR	86.67	73.34	NR	NR	100.00	100.00	NR	NR
Leukopenia grade ≥3 (%)	23.10	39.50	NR	NR	62.00	55.00	NR	NR	20.00	36.60	NR	NR	NR	NR	25.00	12.90	2.70	16.11	NR	NR	NR	NR	13.33	6.67	NR	NR	12.50	0.00	NR	NR
Lower gastrointestinal tract infections any grade (%)	NR	NR	NR	NR	NR	NR	NR	NR	NR	NR	30.00	33.33	NR	NR	NR	NR	NR	NR	NR	NR	NR	NR	NR	NR	NR	NR	NR	NR	NR	NR
Lower gastrointestinal tract infections grade ≥3 (%)	NR	NR	NR	NR	NR	NR	NR	NR	NR	NR	0.00	3.33	NR	NR	NR	NR	NR	NR	NR	NR	NR	NR	NR	NR	NR	NR	NR	NR	NR	NR
Lymphopenia any grade (%)	NR	NR	NR	NR	NR	NR	NR	NR	NR	NR	NR	NR	NR	NR	NR	NR	93.92	99.33	NR	NR	NR	NR	NR	NR	NR	NR	NR	NR	NR	NR
Lymphopenia grade ≥3 (%)	46.20	47.40	NR	NR	NR	NR	NR	NR	NR	NR	NR	NR	NR	NR	NR	NR	72.97	88.59	NR	NR	NR	NR	NR	NR	NR	NR	NR	NR	NR	NR
Middle ear inflammation any grade (%)	NR	NR	NR	NR	4.00	5.00	NR	NR	NR	NR	NR	NR	NR	NR	NR	NR	NR	NR	NR	NR	NR	NR	NR	NR	NR	NR	NR	NR	NR	NR
Middle ear inflammation grade ≥3 (%)	NR	NR	NR	NR	0.00	0.00	NR	NR	NR	NR	NR	NR	NR	NR	NR	NR	NR	NR	NR	NR	NR	NR	NR	NR	NR	NR	NR	NR	NR	NR
Mortality (%)	NR	NR	0.00	8.00	1.60	0.00	0.00	0.00	NR	NR	0.00	0.00	12.50	3.20	3.45	6.45	0.00	0.67	NR	NR	0.00	0.00	NR	NR	5.30	7.58	NR	NR	0.00	3.33
Mucositis any grade (%)	NR	NR	100.00	100.00	93.00	92.00	NR	NR	100.00	100.00	100.00	100.00	95.83	96.70	100.00	100.00	81.76	90.60	NR	NR	NR	NR	100.00	100.00	NR	NR	100.00	100.00	NR	NR
Mucositis grade ≥3 (%)	NR	NR	85.00	92.00	28.00	23.00	NR	NR	53.40	46.70	33.30	40.00	54.10	51.60	32.14	29.03	15.54	18.12	57.00	68.00	70.00	75.90	40.00	60.00	NR	NR	75.00	38.50	NR	NR
Nausea/Vomiting any grade (%)	NR	NR	NR	NR	47.00	67.00	100.00	100.00	100.00	100.00	NR	NR	NR	NR	100.00	100.00	15.54	29.53	39.00	49.00	20.00	34.50	93.37	100.00	NR	NR	100.00	100.00	NR	NR
Nausea/Vomiting grade ≥3 (%)	NR	NR	16.00	31.00	5.00	13.00	7.50	10.70	13.40	20.00	NR	NR	NR	NR	7.10	0.00	1.35	6.71	NR	NR	NR	NR	6.67	20.00	NR	NR	20.80	11.50	20.00	40.00
Neck edema any grade (%)	NR	NR	NR	NR	13.00	16.00	NR	NR	NR	NR	NR	NR	NR	NR	NR	NR	NR	NR	NR	NR	NR	NR	NR	NR	NR	NR	NR	NR	NR	NR
Neck edema grade ≥3 (%)	NR	NR	NR	NR	0.00	1.00	NR	NR	NR	NR	NR	NR	NR	NR	NR	NR	NR	NR	NR	NR	NR	NR	NR	NR	NR	NR	NR	NR	NR	NR
Neuropathy any grade (%)	NR	NR	NR	NR	NR	NR	NR	NR	NR	NR	NR	NR	NR	NR	NR	NR	0.67	0.00	NR	NR	NR	NR	NR	NR	NR	NR	NR	NR	NR	NR
Neuropathy grade ≥3 (%)	NR	NR	NR	NR	NR	NR	NR	NR	NR	NR	NR	NR	NR	NR	NR	NR	0.00	0.00	NR	NR	NR	NR	NR	NR	NR	NR	NR	NR	NR	NR
Neutropenia any grade (%)	NR	NR	23.00	56.00	87.00	92.00	100.00	100.00	30.00	70.00	96.70	100.00	45.83	45.16	100.00	100.00	9.46	30.87	NR	NR	26.70	55.20	NR	NR	NR	NR	100.00	100.00	NR	NR
Neutropenia grade ≥3 (%)	17.90	34.20	NR	NR	35.00	49.00	28.30	16.10	10.00	20.00	33.30	43.30	8.30	3.20	17.90	6.60	1.35	12.75	NR	NR	NR	NR	NR	NR	NR	NR	4.20	0.00	15.00	20.00
Odynophagia any grade (%)	NR	NR	NR	NR	NR	NR	NR	NR	NR	NR	NR	NR	NR	NR	NR	NR	91.89	94.63	NR	NR	NR	NR	NR	NR	NR	NR	NR	NR	NR	NR
Odynophagia grade ≥3 (%)	NR	NR	NR	NR	NR	NR	NR	NR	NR	NR	NR	NR	NR	NR	NR	NR	41.89	51.68	NR	NR	NR	NR	NR	NR	NR	NR	NR	NR	NR	NR
Ototoxicity any grade (%)	NR	NR	NR	NR	NR	NR	NR	NR	NR	NR	NR	NR	NR	NR	NR	NR	NR	NR	15.90	19.50	NR	NR	NR	NR	NR	NR	NR	NR	NR	NR
Ototoxicity grade ≥3 (%)	NR	NR	NR	NR	NR	NR	NR	NR	NR	NR	NR	NR	NR	NR	NR	NR	NR	NR	NR	NR	NR	NR	NR	NR	NR	NR	0.00	0.00	NR	NR

Peripheral sensory neuropathy any grade (%)	NR	NR	NR	NR	0.00	5.00	NR	NR	NR	NR	NR	NR	NR	NR	NR	NR	NR	NR	NR	NR	NR	NR	NR	NR	NR	NR	NR	NR	NR	NR
Peripheral sensory neuropathy grade ≥3 (%)	NR	NR	NR	NR	0.00	0.00	NR	NR	NR	NR	NR	NR	NR	NR	NR	NR	NR	NR	NR	NR	NR	NR	NR	NR	NR	NR	NR	NR	NR	NR
Pharyngitis any grade (%)	NR	NR	NR	NR	NR	NR	NR	NR	NR	NR	NR	NR	NR	NR	NR	NR	NR	NR	NR	NR	NR	NR	NR	NR	NR	NR	100.00	100.00	NR	NR
Pharyngitis grade ≥3 (%)	NR	NR	NR	NR	NR	NR	NR	NR	NR	NR	NR	NR	NR	NR	NR	NR	NR	NR	NR	NR	NR	NR	NR	NR	NR	NR	54.20	53.80	NR	NR
Rash any grade (%)	NR	NR	NR	NR	NR	NR	100.00	100.00	NR	NR	NR	NR	NR	NR	NR	NR	NR	NR	NR	NR	NR	NR	NR	NR	NR	NR	NR	NR	NR	NR
Rash grade ≥3 (%)	NR	NR	NR	NR	NR	NR	0.00	1.80	NR	NR	NR	NR	NR	NR	NR	NR	NR	NR	NR	NR	NR	NR	NR	NR	NR	NR	NR	NR	NR	NR
Renal dysfunction any grade (%)	NR	NR	NR	NR	NR	NR	NR	NR	NR	NR	NR	NR	NR	NR	NR	NR	0.00	0.67	NR	NR	NR	NR	NR	NR	NR	NR	NR	NR	NR	NR
Renal dysfunction grade ≥3 (%)	NR	NR	NR	NR	NR	NR	NR	NR	NR	NR	NR	NR	NR	NR	NR	NR	0.00	0.00	NR	NR	NR	NR	NR	NR	NR	NR	NR	NR	NR	NR
Renal toxicity any grade (%)	NR	NR	NR	NR	NR	NR	NR	NR	NR	NR	3.33	6.67	16.67	9.68	0.00	0.00	NR	NR	NR	NR	NR	NR	NR	NR	NR	NR	4.17	3.85	NR	NR
Renal toxicity grade ≥3 (%)	NR	NR	NR	NR	NR	NR	0.00	0.00	NR	NR	0.00	0.00	0.00	0.00	0.00	0.00	NR	NR	NR	NR	NR	NR	NR	NR	NR	NR	0.00	0.00	5.00	16.60
Renal toxicity not specified (%)	NR	NR	31.00	48.00	NR	NR	NR	NR	NR	NR	NR	NR	NR	NR	NR	NR	NR	NR	36.00	83.00	26.70	31.00	NR	NR	NR	NR	NR	NR	NR	NR
Salivary duct inflammation any grade (%)	NR	NR	NR	NR	13.00	11.00	NR	NR	NR	NR	NR	NR	NR	NR	NR	NR	NR	NR	NR	NR	NR	NR	NR	NR	NR	NR	NR	NR	NR	NR
Salivary duct inflammation grade ≥3 (%)	NR	NR	NR	NR	2.00	0.00	NR	NR	NR	NR	NR	NR	NR	NR	NR	NR	NR	NR	NR	NR	NR	NR	NR	NR	NR	NR	NR	NR	NR	NR
Stomatitis any grade (%)	NR	NR	NR	NR	NR	NR	100.00	100.00	NR	NR	NR	NR	NR	NR	NR	NR	NR	NR	NR	NR	NR	NR	NR	NR	NR	NR	100.00	100.00	NR	NR
Stomatitis grade ≥3 (%)	NR	NR	NR	NR	NR	NR	15.10	12.50	NR	NR	NR	NR	NR	NR	NR	NR	NR	NR	NR	NR	NR	NR	NR	NR	NR	NR	54.20	53.80	25.00	23.30
Thrombocytopenia any grade (%)	NR	NR	NR	NR	84.00	66.00	90.00	100.00	16.60	23.30	70.00	83.30	8.20	3.20	100.00	100.00	2.70	2.70	23.00	41.00	6.66	10.30	NR	NR	NR	NR	100.00	100.00	NR	NR
Thrombocytopenia grade ≥3 (%)	5.10	5.30	NR	NR	3.00	2.00	7.50	1.80	3.30	10.00	0.00	6.70	0.00	0.00	0.00	3.30	2.70	2.01	NR	NR	NR	NR	NR	NR	NR	NR	0.00	0.00	5.00	16.60
Tinnitus any grade (%)	NR	NR	NR	NR	5.00	25.00	NR	NR	NR	NR	NR	NR	NR	NR	NR	NR	NR	NR	NR	NR	NR	NR	NR	NR	NR	NR	NR	NR	NR	NR
Tinnitus grade ≥3 (%)	NR	NR	NR	NR	0.00	0.00	NR	NR	NR	NR	NR	NR	NR	NR	NR	NR	NR	NR	NR	NR	NR	NR	NR	NR	NR	NR	NR	NR	NR	NR
Transaminase elevation any grade (%)	NR	NR	NR	NR	NR	NR	NR	NR	NR	NR	NR	NR	NR	NR	NR	NR	4.73	14.77	NR	NR	NR	NR	NR	NR	NR	NR	NR	NR	NR	NR
Transaminase elevation grade ≥3 (%)	NR	NR	NR	NR	NR	NR	NR	NR	NR	NR	NR	NR	NR	NR	NR	NR	1.35	5.37	NR	NR	NR	NR	NR	NR	NR	NR	NR	NR	NR	NR
Upper gastrointestinal tract infections any grade (%)	NR	NR	NR	NR	NR	NR	NR	NR	NR	NR	100.00	100.00	NR	NR	NR	NR	NR	NR	NR	NR	NR	NR	NR	NR	NR	NR	NR	NR	NR	NR
Upper gastrointestinal tract infections grade ≥3 (%)	NR	NR	NR	NR	NR	NR	NR	NR	NR	NR	20.00	16.67	NR	NR	NR	NR	NR	NR	NR	NR	NR	NR	NR	NR	NR	NR	NR	NR	NR	NR
Weight loss ≥10% (%)	43.60	42.10	31.00	40.00	NR	NR	NR	NR	NR	NR	NR	NR	NR	NR	NR	NR	11.49	17.45	NR	NR	23.30	41.40	NR	NR	NR	NR	NR	NR	NR	NR
Xerostomia any grade (%)	NR	NR	NR	NR	66.00	63.00	NR	NR	100.00	100.00	NR	NR	NR	NR	NR	NR	48.65	51.68	NR	NR	NR	NR	NR	NR	NR	NR	NR	NR	NR	NR
Xerostomia grade ≥3 (%)	NR	NR	NR	NR	2.00	1.00	NR	NR	16.70	20.00	NR	NR	NR	NR	NR	NR	0.00	0.67	NR	NR	NR	NR	NR	NR	NR	NR	NR	NR	NR	NR

Table S3 Long term adverse events in the studies meeting inclusion criteria. Studies that did not report any long term adverse events are not presented. Abbreviations: n, number of patients assessed for safety; NR, not reported.

	Chaturvedi A, 2024		Kiyota N, 2022		Mitra D, 2011		Noronha V, 2017	
Long term toxicity	Weekly (n=26)	3-weekly (n=25)	Weekly (n=121)	3-weekly (n=129)	Weekly (n=25)	3-weekly (n=26)	Weekly (n=116)	3-weekly (n=126)
Alopecia any grade (%)	NR	NR	9.00	5.00	NR	NR	NR	NR
Alopecia grade ≥3 (%)	NR	NR	NR	NR	NR	NR	NR	NR
Any chronic toxicity grade ≥3 (%)	NR	NR	NR	NR	NR	NR	10.34	13.49
Dental caries any grade (%)	NR	NR	5.00	2.00	NR	NR	NR	NR
Dental caries grade ≥3 (%)	NR	NR	0.00	1.00	NR	NR	NR	NR
Dermatitis any grade (%)	NR	NR	17.00	18.00	NR	NR	13.79	14.29
Dermatitis grade ≥3 (%)	NR	NR	0.00	0.00	NR	NR	0.00	0.00
Dry skin any grade (%)	NR	NR	5.00	5.00	NR	NR	NR	NR
Dry skin grade ≥3 (%)	NR	NR	0.00	0.00	NR	NR	NR	NR
Dysgeusia any grade (%)	NR	NR	60.00	57.00	NR	NR	19.83	22.22
Dysgeusia grade ≥3 (%)	NR	NR	NR	NR	NR	NR	0.00	0.00
Dysphagia any grade (%)	14.00	20.00	39.00	35.00	NR	NR	14.66	10.32
Dysphagia grade ≥3 (%)	NR	NR	NR	NR	NR	NR	3.45	3.97
Fatigue any grade (%)	NR	NR	18.00	12.00	NR	NR	NR	NR
Fatigue grade ≥3 (%)	NR	NR	0.00	1.00	NR	NR	NR	NR
Gum infection any grade (%)	NR	NR	3.00	2.00	NR	NR	NR	NR
Gum infection grade ≥3 (%)	NR	NR	0.00	0.00	NR	NR	NR	NR
Hearing impairment any grade (%)	NR	NR	14.00	16.00	NR	NR	NR	NR
Hearing impairment grade ≥3 (%)	NR	NR	3.00	0.00	NR	NR	NR	NR
Hearing loss any grade (%)	NR	NR	NR	NR	NR	NR	7.76	28.57
Hearing loss grade ≥3 (%)	NR	NR	NR	NR	NR	NR	4.31	15.87
Hoarseness any grade (%)	NR	NR	2.00	2.00	NR	NR	NR	NR
Hoarseness grade ≥3 (%)	NR	NR	0.00	0.00	NR	NR	NR	NR
Hypertriglicerydemia any grade (%)	NR	NR	NR	NR	NR	NR	4.31	1.59
Hypertriglicerydemia grade ≥3 (%)	NR	NR	NR	NR	NR	NR	1.72	0.79

Hypothyroidism any grade (%)	0.00	0.00	34.00	25.00	NR	NR	30.17	28.57
Hypothyroidism grade ≥3 (%)	NR	NR	0.00	0.00	NR	NR	0.00	0.00
Infection any grade (%)	NR	NR	NR	NR	NR	NR	17.24	22.22
Infection grade ≥3 (%)	NR	NR	NR	NR	NR	NR	5.17	4.76
Laryngeal edema any grade (%)	NR	NR	9.00	11.00	8.00	3.85	NR	NR
Laryngeal edema grade ≥3 (%)	NR	NR	1.00	0.00	NR	NR	NR	NR
Larynx mucositis any grade (%)	NR	NR	6.00	3.00	NR	NR	NR	NR
Larynx mucositis grade ≥3 (%)	NR	NR	0.00	0.00	NR	NR	NR	NR
Lung infection any grade (%)	NR	NR	2.00	2.00	NR	NR	NR	NR
Lung infection grade ≥3 (%)	NR	NR	1.00	0.00	NR	NR	NR	NR
Middle ear inflammation any grade (%)	NR	NR	4.00	6.00	NR	NR	NR	NR
Middle ear inflammation grade ≥3 (%)	NR	NR	0.00	0.00	NR	NR	NR	NR
Mucositis any grade (%)	NR	NR	NR	NR	NR	NR	5.17	3.97
Mucositis grade ≥3 (%)	NR	NR	NR	NR	NR	NR	0.00	0.79
Nausea any grade (%)	NR	NR	4.00	1.00	NR	NR	NR	NR
Nausea grade ≥3 (%)	NR	NR	0.00	0.00	NR	NR	NR	NR
Neck edema any grade (%)	NR	NR	22.00	25.00	16.00	11.54	NR	NR
Neck edema grade ≥3 (%)	NR	NR	2.00	0.00	NR	NR	NR	NR
Neck soft tissue necrosis any grade (%)	NR	NR	3.00	1.00	NR	NR	NR	NR
Neck soft tissue necrosis grade ≥3 (%)	NR	NR	1.00	0.00	NR	NR	NR	NR
Odynophagia any grade (%)	NR	NR	NR	NR	NR	NR	10.34	8.73
Odynophagia grade ≥3 (%)	NR	NR	NR	NR	NR	NR	1.72	3.97
Oral mucositis any grade (%)	NR	NR	17.00	14.00	NR	NR	NR	NR
Oral mucositis grade ≥3 (%)	NR	NR	0.00	1.00	NR	NR	NR	NR
Osteonecrosis any grade (%)	0.00	0.00	NR	NR	NR	NR	NR	NR
Osteonecrosis of jaw any grade (%)	NR	NR	2.00	3.00	NR	NR	NR	NR
Osteonecrosis of jaw grade ≥3 (%)	NR	NR	0.00	1.00	NR	NR	NR	NR
Ototoxicity any grade(%)	7.00	0.00	4.00	3.00	NR	NR	NR	NR
Overall late any grade toxicities (%)	28.50	26.60	NR	NR	NR	NR	NR	NR

Peripheral motor neuropathy any grade (%)	NR	NR	3.00	2.00	NR	NR	NR	NR
Peripheral motor neuropathy grade ≥3 (%)	NR	NR	0.00	0.00	NR	NR	NR	NR
Peripheral sensory neuropathy any grade (%)	NR	NR	8.00	10.00	NR	NR	NR	NR
Peripheral sensory neuropathy grade ≥3 (%)	NR	NR	0.00	0.00	NR	NR	NR	NR
Pharynx mucositis any grade (%)	NR	NR	12.00	9.00	NR	NR	NR	NR
Pharynx mucositis grade ≥3 (%)	NR	NR	1.00	0.00	NR	NR	NR	NR
Renal toxicity any grade (%)	0.00	0.00	NR	NR	NR	NR	NR	NR
Salivary duct inflammation any grade (%)	NR	NR	7.00	6.00	NR	NR	NR	NR
Salivary duct inflammation grade ≥3 (%)	NR	NR	0.00	0.00	NR	NR	NR	NR
Skin atrophy any grade (%)	NR	NR	3.00	2.00	NR	NR	NR	NR
Skin atrophy grade ≥3 (%)	NR	NR	0.00	0.00	NR	NR	NR	NR
Subcutaneous toxicity any grade (%)	NR	NR	NR	NR	NR	NR	56.90	52.38
Subcutaneous toxicity grade ≥3 (%)	NR	NR	NR	NR	NR	NR	0.00	1.59
Thromboembolic event any grade (%)	NR	NR	NR	NR	NR	NR	0.86	0.00
Thromboembolic event grade ≥3 (%)	NR	NR	NR	NR	NR	NR	0.00	0.00
Tinnitus any grade (%)	NR	NR	7.00	15.00	NR	NR	NR	NR
Tinnitus grade ≥3 (%)	NR	NR	0.00	0.00	NR	NR	NR	NR
Trismus any grade (%)	NR	NR	7.00	3.00	NR	NR	8.62	7.14
Trismus grade ≥3 (%)	NR	NR	1.00	0.00	NR	NR	1.72	0.00
Xerostomia any grade (%)	14.00	6.00	60.00	59.00	24.00	19.23	55.17	53.97
Xerostomia grade ≥3 (%)	NR	NR	0.00	0.00	NR	NR	0.86	0.79
